# The Versatility of the Free Vastus Lateralis Muscle Flap: Orbital Reconstruction After Removal of Complex Vascular Malformation in a Pediatric Patient

**DOI:** 10.3389/fped.2021.703330

**Published:** 2021-08-19

**Authors:** Francesca Grussu, Luigino Santecchia, Urbano Urbani, Giorgio Spuntarelli, Massimo Rollo, May El Hachem, Antonino Romanzo, Mario Zama

**Affiliations:** ^1^Plastic and Maxillofacial Surgery Unit, Bambino Gesù Children Hospital (IRCCS), Rome, Vatican City; ^2^Orthopaedic Unit of Palidoro, Bambino Gesù Children Hospital (IRCCS), Rome, Vatican City; ^3^Interventional Radiology Unit, Bambino Gesù Children Hospital (IRCCS), Rome, Vatican City; ^4^Dermatology Unit, Genetics and Rare Disease Research Division, Bambino Gesù Children Hospital (IRCCS), Rome, Vatican City; ^5^Genodermatosis Unit, Genetics and Rare Disease Research Division, Bambino Gesù Children Hospital (IRCCS), Rome, Vatican City; ^6^Ophtalmology Unit, Bambino Gesù Children Hospital (IRCCS), Rome, Vatican City

**Keywords:** vascular orbital lesions, orbital reconstruction, pediatric microsurgery, vastus lateralis free flap, pediatric orbital reconstruction, vascular malformation

## Abstract

**Introduction:** Vascular orbital lesions in pediatric population represent a demanding therapeutic challenge which requires a multidisciplinary team. In severe cases, orbital enucleation can be considered. Surgical management of enucleated orbital region in children, differently from the adults, represents a challenging procedure owing to the intrinsic relation between volume replacement and normal orbital growth. Many reconstructive options have been proposed, and many donor sites have been utilized for this purpose but each one have demonstrated potential disadvantages. Despite its well-known versatility, no report of the vastus lateralis free flap in children requiring orbital reconstruction exists in literature. Herein, we propose this surgical strategy as a valid option for the reconstruction of an extended orbital defect in a pediatric patient suffering from a mixed type of vascular malformation.

**Material and Methods:** A patient was referred from a foreign country with an unclear medical history, presenting exorbitism and exophthalmos, proptosis of the eyeball, visus 4/10, and limited ocular motility. We made clinical-instrumental investigations with a diagnosis of complex vascular malformation. It expanded in intraorbital and retrorbital space with bulb anterior dislocation and optic nerve involvement. We performed an emptying of the orbital content *via* transconjunctival and *via* coronal incision with eyelid preservation. A free vastus lateralis muscle flap was used for reconstruction, filling the orbital cavity. We anastomosed the flap on the superficial temporal artery. An ocular conformator was then positioned.

**Results:** We report the result at 12 months, showing a good orbital rehabilitation with an adequate prosthetic cavity, a good recovery of volume and facial symmetry, guaranteeing balanced orbital and periorbital growth. There were no major or minor complications associated with the procedure.

**Discussion:** The reconstruction of the orbit remains a “surgical challenge” both in adults, whose goal is the restoration of volume, adequate symmetry and facial esthetics, and children, in which correcting the asymmetry has the additional objective to balance orbital growth. Many reconstructive techniques have been proposed, including the use of free flaps. The versatility of the free vastus lateralis muscle flap is well-known. It offers adequate amount of tissue with minimal morbidity to the donor site, provides a long pedicle, gives the possibility of simultaneous work in a double team, and has a constant anatomy and a safe and rapid dissection. There are no descriptions of its use for pediatric orbital reconstructions.

**Conclusions:** In our opinion, the free vastus lateralis flap should be included as one of the best option for orbital pediatric reconstruction after enucleation.

## Introduction

The reported incidence of vascular orbital lesions in pediatric population ranges from 5.5 to 22% of all orbital lesions (1–6). Orbital venous malformation (OVM) ranks as the most common type of vascular malformation in the orbit. OVMs belong to the low-flow category and can be purely venous or mixed. They are usually not noticed at birth but become clinically obvious in the 1st decade causing prominent proptosis, severe globe displacement, deep orbital pain, disfigurement, even affecting vision development and ocular motility and acuity (7–9). They represent a demanding therapeutic challenge which requires a multidisciplinary team including dermatologists, interventional radiologists, oculoplastic surgeons, and plastic surgeons. Accurate diagnosis and localization of these lesions are essential for proper management to avoid serious complications. There is no current consensus on the first-line treatment for OVM and both surgical and non-surgical approaches have been proposed (10–14). Roughly, 50% can be managed conservatively once the diagnosis has been made (7). Medical management is recommended and useful in different cases; nevertheless, in the presence of optic neuropathy, owing to optic nerve compression or stretching, anisometropy or significant cosmetic deformity surgery is indicated. In severe cases, when an orbital lymphatic-venous malformation is localized in the posterior orbit presenting with a blind and painful eye, orbital exenteration can be considered even in the pediatric population (11). Surgical management of enucleated orbital region in children, differently from the adults, represents a demanding procedure because of the intrinsic relation between volume replacement and normal orbital growth. Many reconstructive options have been proposed, and pedicled muscle flaps derived from the temporalis and pectoralis major have been used. Unfortunately, their restricted arc of rotation, due to their short vascular pedicles, limits the volume of tissue effectively transferred to the orbit (15, 16). Thanks to the recent knowledge in pediatric microsurgery, these limitations have been largely overcome: a free microvascular flap transfer can provide the desired tissue composition and volume and an aesthetically pleasing reconstruction. Many donor muscles, rectus abdominis, latissimus dorsi, serratus anterior, and gracilis muscle, have been utilized for this purpose, but each one have demonstrated potential disadvantages. Despite its well-known versatility, no report of the vastus lateralis free flap in children requiring orbital reconstruction exists in literature. Herein, we propose this surgical strategy as a valid option for the reconstruction of an extended orbital defect in a pediatric patient suffering from a mixed type of vascular malformation.

## Case Description

**Figure 1:**
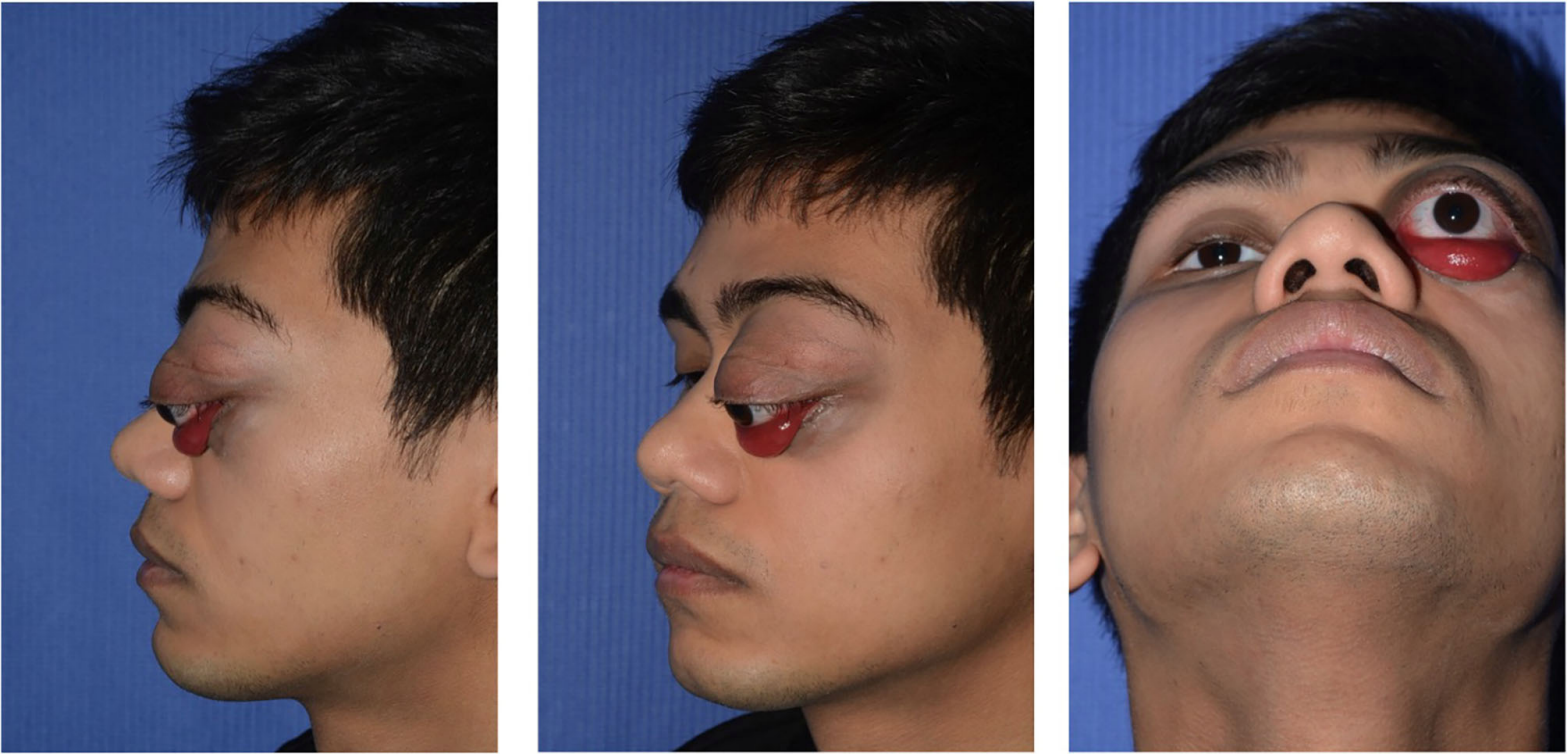
A 13-year-old Indian boy was referred to our ophthalmic pediatric clinic for the evaluation of a mass in the left eye, marked exorbitism, exophthalmos, proptosis, and chemosis.

**Figure 2:**
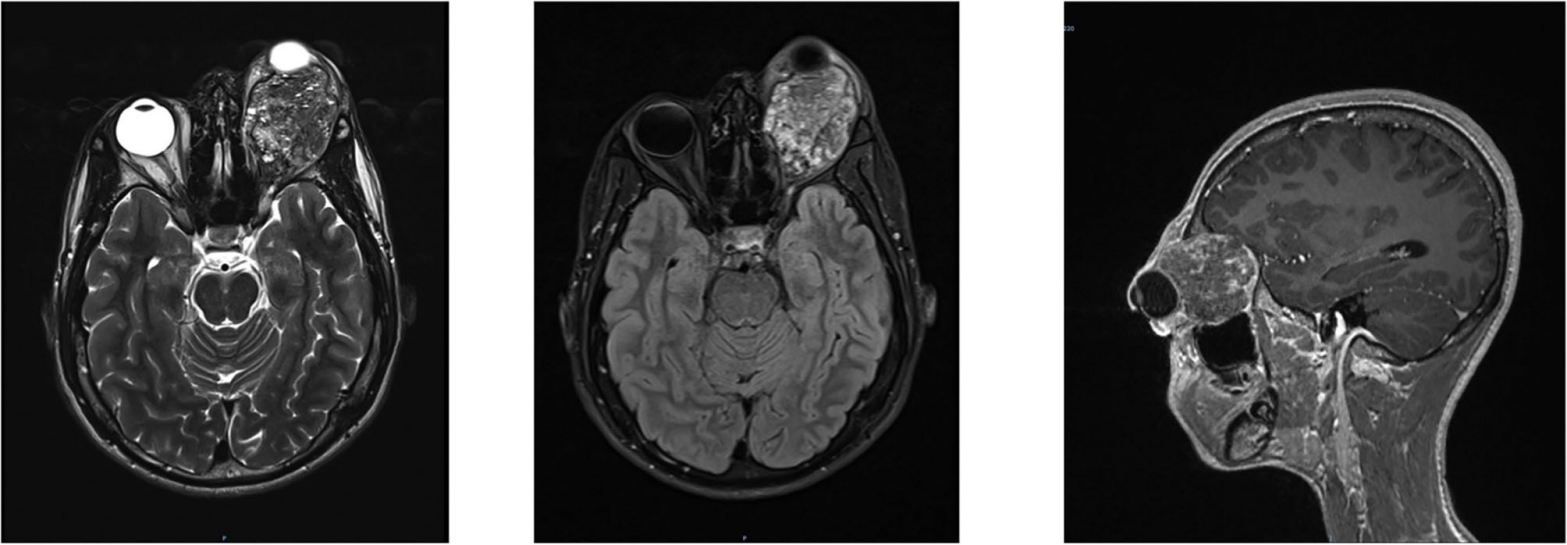
Magnetic resonance imaging of brain and orbits, with T1- and T2-weighted sequences, showed the presence of a bulky pathological tissue located in the left retrobulbar intraorbital space, displacing anteriorly the eyeball and incorporating the optic nerve, which did not appear recognizable in all its intraorbital segments.

**Figure 3:**
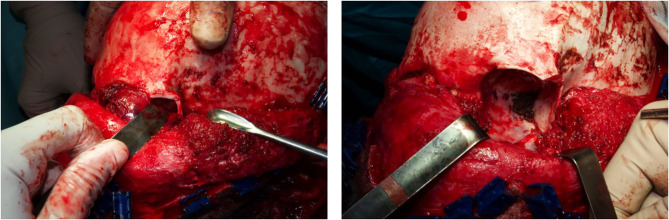
The orbital cavity before and after the removal of the mass with all orbital content, *via* coronal approach, after left malar body and arch exposition.

**Figure 4:**
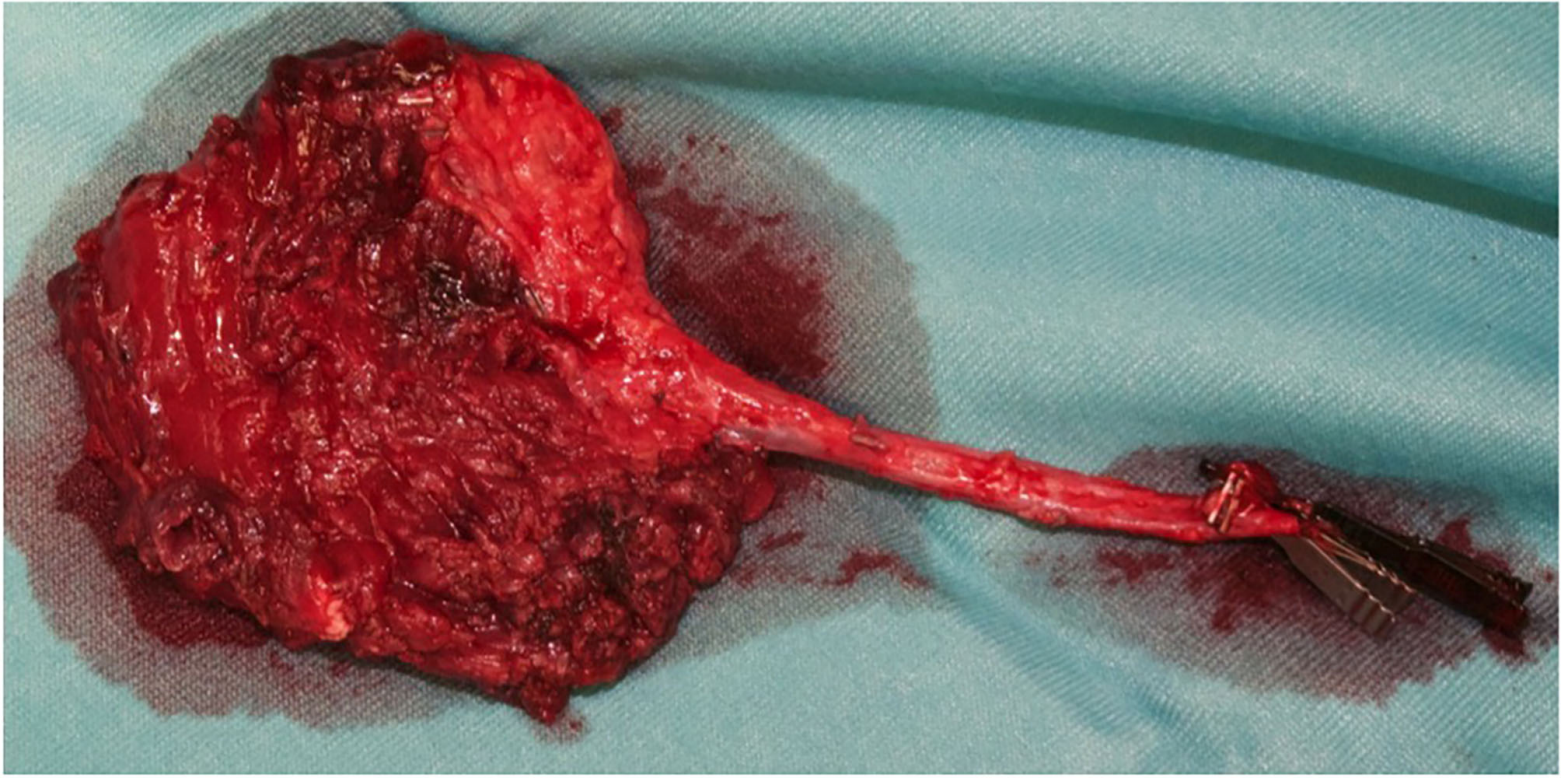
The free vastus lateralis flap harvested through a muscle sparing technique.

**Figure 5:**
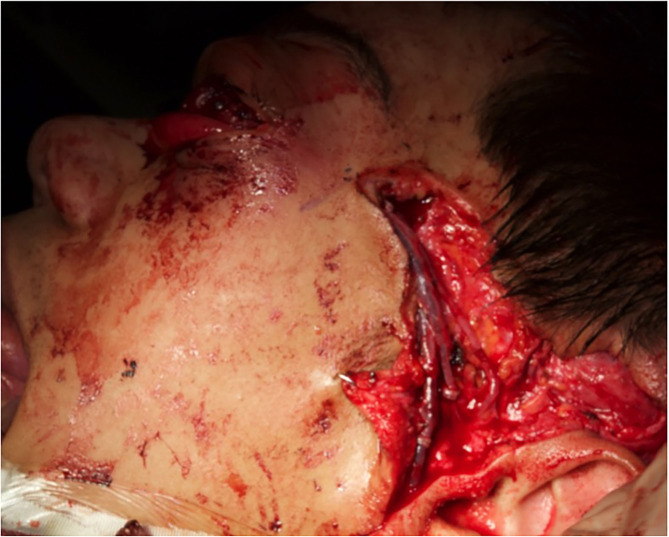
The vastus lateralis free flap harvested with a muscle sparing technique and its inset. Note the pedicle, anastomosed on the temporal vessels, passed laterally to the external orbital frame through an incisura and brought to the temporal area through a subcutaneous tunnel.

**Figure 6:**
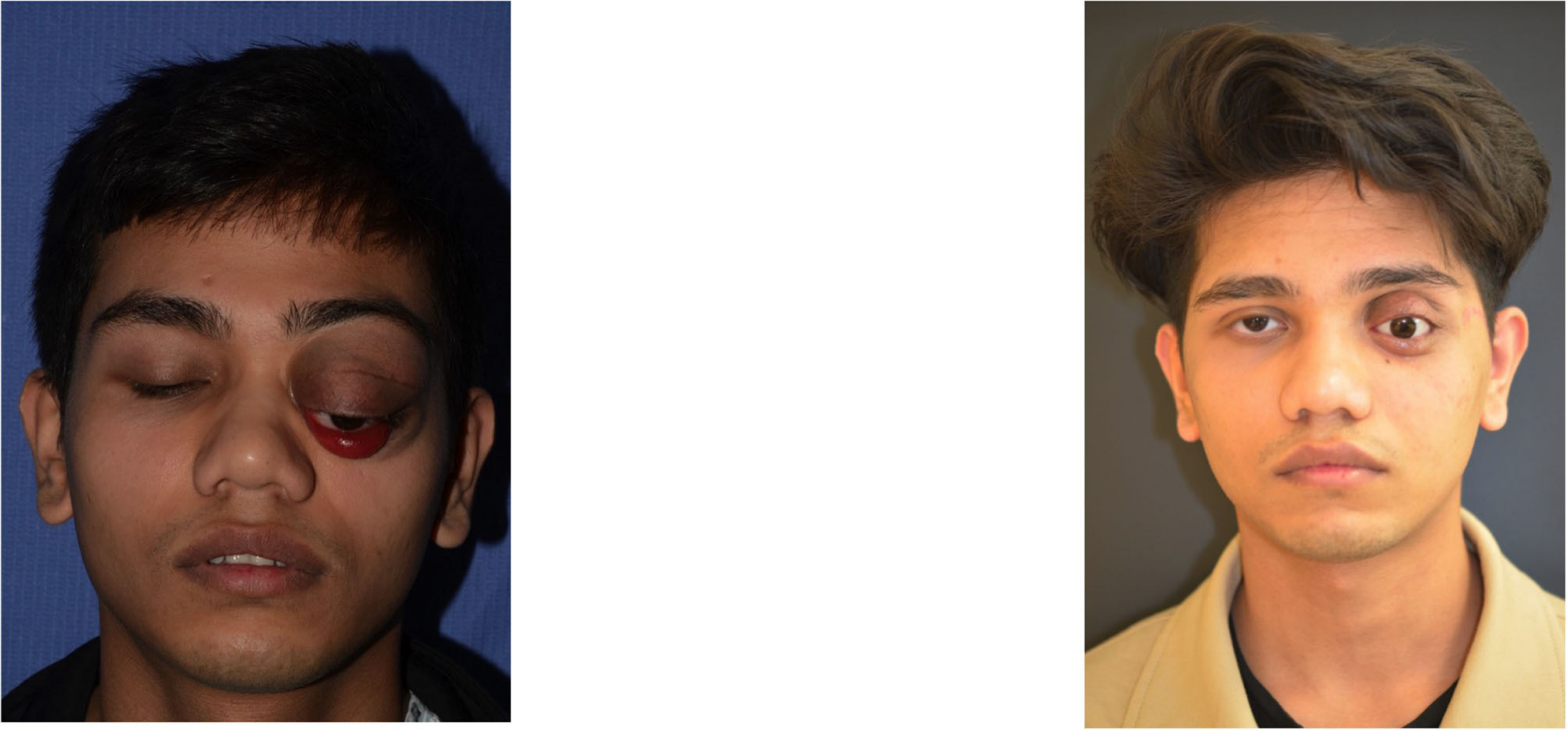
We obtain a good orbital rehabilitation with an adequate prosthetic cavity, a good volume recovery, and therefore a satisfactory facial symmetry.

A 13-year-old Indian boy was referred to our ophthalmic pediatric clinic for the evaluation of a mass in the left eye with an unclear medical history (Figure 1). There was no positive family history of hemangiomas or vascular malformations. Moreover, there was no history of trauma or focal neurological deficits. The ophthalmological evaluation revealed no light perception on the left, with a red, dilated pupil and a positive reverse relative afferent pupillary defect. The patient exhibited marked exorbitism, exophthalmos, proptosis, and chemosis. Extraocular movements were limited in all directions. Visual acuity was 4/10 −1. The fundus examination was normal. Findings from the right-eye examination were normal with visual acuity of 7/10. Magnetic resonance imaging (MRI) of the brain and orbits (with T1 and T2 weighted sequences) with angiographic sequences (MRA) showed the presence of a bulky pathological tissue located in the left retrobulbar intraorbital space, characterized by a T2 inhomogeneous hyperintense signal (owing to the presence of some minute calcifications recognizable in the CT scan) and by inhomogeneous postcontrast enhancement phenomena (Figure 2). The tissue totally occupied the orbital cavity (which appeared enlarged), displacing anteriorly the eyeball and incorporating the optic nerve, which did not appear recognizable in all its intraorbital segments. The extrinsic ocular muscles were stretched. The patient underwent diagnostic angiography which revealed tenuous and modest contrast impregnation of about 3 ×1.5 cm in the site of the retrobulbar expansive formation, supported exclusively by intraorbital branches of the ophthalmic artery. Arterial afferents from the ipsilateral external carotid system, particularly from the internal maxillary and facial arteries, were not appreciated. Furthermore, the exam documented the absence of early venous drainage, with venous discharge to the ophthalmic vein system and cavernous sinus and small persistent venous ectasias in late venous phases. Based on these descriptions and on biopsy result, a complex mixed venous vascular malformation was diagnosed. Thus, after a multidisciplinary meeting and based on the worsening clinic presentation, we planned the surgical removal of the lymphatic-venous malformation. We firstly performed a transconjunctival emptying of the orbital content with extrinsic orbicular muscle demarcation and preliminary skeletonization of the orbital mass for about 3 cm in depth. *Via* coronal approach, after left malar body and arch exposition, we then dissected up to the orbital apex and thus macroscopically removed the mass with all orbital content (Figure 3). Contextually, a vastus lateralis free flap was harvested, tailored to fit the defect, with regard to the pedicle length and muscle width, length, and depth. We performed a muscle-sparing harvesting, leaving the remaining muscular portion innervated and functional (Figure 4). The flap was then set inside the cavity, and the pedicle passed laterally to the external orbital frame through an incisura and brought to the temporal area through a subcutaneous tunnel. It was anastomosed on the superficial temporal artery (Figure 5). Donor site was closed primarily. An ocular conformer was positioned and during the follow-up fitted to the expected and physiologic muscle atrophy. At 1 year follow-up, our patient showed a good orbital rehabilitation with an adequate prosthetic cavity, a good volume recovery, and, therefore, a satisfactory facial symmetry (Figure 6). No morbidity at the donor site and no recurrence of malformation were recorded.

## Discussion

For a correct management of orbital vascular malformations, a deep comprehension of their anatomic presentation and classification is mandatory. The ISSVA classification system ranks orbital lymphatic-venous malformations as low-flow vascular malformations, which can be purely venous or mixed, presenting a variable venous component with a variable systemic venous out-flow (1, 2). In terms of location, these malformations can be superficial (anterior, visible lesions limited to the conjunctiva or eyelid), deep (retrobulbar or peribulbar lesions without exterior manifestation), combined (superficial and deep), and complex (involving periorbital and intracranial tissues) (4). They represent 1–3% of all orbital masses being generally present at birth but do not clinically manifest until the first decade of life, tending to grow proportionally to the growth of the child (1, 6). They may appear after an upper respiratory tract infection, trauma, or with acute intralesional bleeding. Depending on their location, size, and relationships to adjacent tissues, they can cause structural and functional impairments such as proptosis, diplopia, swelling of periorbital soft tissue, vision loss, or periodic ecchymosis secondary to hemorrhage and thrombosis. Other presentations can include extraocular motility restriction, physical disfigurement, and compressive optic neuropathy (4). Due to the lack of consensus on therapy for orbital lymphatic-venous malformations and to their proximity to vital structures, their treatment includes a large array of approaches. Observation is recommended for patients without significant symptoms or cosmetic issues, while a complete eradication is difficult owing to a high risk of morbidity and recurrence; therefore, it is generally advisable to opt for monitoring the lesions until any significant symptoms occur (4–7). Sclerosing therapy including ethanol, OK-432, sodium tetradecyl sulfate (Sotradecol, Mylan), morrhuate sodium, doxycycline, bleomycin, and pinyangmycin are non-surgical strategies adopted for decades (1, 4, 5). Carbon dioxide laser, beta irradiation, and adjunctive systemic corticosteroids are other treatment options (1). Recently, systemic sildenafil for the treatment of microcystic lesions and sirolimus has shown positive results (1, 8, 9). As recurrence and revascularization of residual lesions are common, OVM surgical management is controversial. Furthermore, surgery carries a risk of damaging normal structures depending on location and extent of the lesion (10–12). Taking into account the relationships between orbital structures and vascular dynamics, the surgical approach should be tailored for each lesion (13). In severe cases, when extensive deep orbital lymphatic-venous malformation cause blindness and eye pain together with severe cosmetic disfigurement, orbital exenteration can be considered (4–14). Reconstruction of the enucleate pediatric orbit poses a different set of challenges both for the psychological effects on child and parents and for the long-term orbital growth. An anophthalmic eye can lead to severely underdeveloped bony orbital growth thus affecting the overall facial development. Keep in mind the phases of orbital development is of paramount importance in managing the anophthalmic orbit. The normal infant eye is ~70% of its adult size and grows most rapidly in the first 12 months of age (15). At 3 months of age, the face is only ~40% the size of the adult face, and by 2 years of age, the face reaches 70% of the adult size. By 5.5 years of age, the pediatric face is ~90% of the adult dimensions (16). The known significant decrease in orbital growth of the orbit after enucleation is most marked when it is performed before the age of 13 years. Whereas, when enucleation is performed after the age of 12 years, for several years little differences in the orbital measures are recorded, but later compensatory enlargement of the periorbital paranasal sinuses occurs (17). Hence, orbital soft tissue volume is a critical factor in orbital bone development and its replacement is essential to maintain the normal orbital and facial bone growth. Generally, the management of an anophthalmic orbit in a child younger than 5 years requires an implant that can increase in size, such as a dermis-fat graft or orbital tissue expander. A large fixed-sized orbital implant can be placed in children older than 5 years of age (18). Many reconstructive options have been proposed, and pedicle muscle flaps, such as the temporalis and pectoralis major flap, have been used. Unfortunately, their restricted arc of rotation, owing to their short vascular pedicles, limits the volume of tissue effectively transferable to the orbit (19, 20). In addition, the pectoralis flap may be associated with excessive bulk in neck and remarkable donor site deformity with undesirable jaw movement changes related to the transposition of the temporalis flap. Thanks to the recent knowledge in pediatric microsurgery, these limitations have been largely overcome. A free microvascular flap reconstruction provides larger and well-vascularized tissue and greater adaptability avoiding orientation issues (19, 20). Many donor muscles, due to their reliable anatomy, have been utilized for this purpose, but each one has demonstrated potential disadvantages. Among these, the latissimus dorsi flap, despite its suitable volume and pedicle length, requires intraoperative repositioning, preventing simultaneous flap harvesting and enucleation (21). The main disadvantage of the serratus anterior is its inadequate volume while gracilis muscle has a relatively short pedicle. The rectus abdominis flap, although it provides an excellent pedicle caliber, a large volume of muscle and the chance for simultaneous flap harvest, it disrupts the abdominal wall integrity, being potentially associated with development of abdominal hernias (22). In our experience, the vastus lateralis flap overcomes each of the disadvantages of the most common flaps, presenting the following advantages: (1) It has consistent anatomy, which allows for a simple dissection from the lateral thigh (23–25). (2) It provides an adequate and adaptable amount of tissue, leaving the remaining muscular portion innervated and functional thanks to the muscle sparing harvesting technique. (3) It has no donor site morbidity (26–28). (4) The flap can be harvested with the patient in a supine position, allowing surgical demolition and reconstruction simultaneity. (5) It allows immediate ocular prosthetic placement and therefore a good aesthetic result in one-stage procedure. Based on such a favorable characteristics, in our opinion, this versatile flap should be included as one of the best option for orbital pediatric reconstruction after enucleation.

## Data Availability Statement

The original contributions presented in the study are included in the article/supplementary material, further inquiries can be directed to the corresponding author/s.

## Ethics Statement

Ethical review and approval was not required for the study on human participants in accordance with the local legislation and institutional requirements. Written informed consent to participate in this study was provided by the participants' legal guardian/next of kin. Written informed consent was obtained from the individual(s), and minor(s)' legal guardian/next of kin, for the publication of any potentially identifiable images or data included in this article.

## Author Contributions

All authors listed have made a substantial, direct and intellectual contribution to the work, and approved it for publication.

## Conflict of Interest

The authors declare that the research was conducted in the absence of any commercial or financial relationships that could be construed as a potential conflict of interest.

## Publisher's Note

All claims expressed in this article are solely those of the authors and do not necessarily represent those of their affiliated organizations, or those of the publisher, the editors and the reviewers. Any product that may be evaluated in this article, or claim that may be made by its manufacturer, is not guaranteed or endorsed by the publisher.
